# Doxycycline, Azithromycin and Vitamin C (DAV): A potent combination therapy for targeting mitochondria and eradicating cancer stem cells (CSCs)

**DOI:** 10.18632/aging.101905

**Published:** 2019-04-19

**Authors:** Marco Fiorillo, Fanni Tóth, Federica Sotgia, Michael P. Lisanti

**Affiliations:** 1 Translational Medicine, School of Environment and Life Sciences, Biomedical Research Centre (BRC), University of Salford, Greater Manchester, M5 4WT, United Kingdom; 2 The Department of Pharmacy, Health and Nutritional Sciences, The University of Calabria, Cosenza, Italy

**Keywords:** Doxycycline, Azithromycin, Vitamin C, combination therapy, mitochondrial ATP depletion, glycolysis

## Abstract

Here, we devised a new strategy for eradicating cancer stem cells (CSCs), via a “synthetic-metabolic” approach, involving two FDA-approved antibiotics and a dietary vitamin supplement. This approach was designed to induce a “rho-zero-like” phenotype in cancer cells. This strategy effectively results in the synergistic eradication of CSCs, using vanishingly small quantities of two antibiotics. The 2 metabolic targets are i) the large mitochondrial ribosome and ii) the small mitochondrial ribosome. Azithromycin inhibits the large mitochondrial ribosome as an off-target side-effect. In addition, Doxycycline inhibits the small mitochondrial ribosome as an off-target side-effect. Vitamin C acts as a mild pro-oxidant, which can produce free radicals and, as a consequence, induces mitochondrial biogenesis. Remarkably, treatment with a combination of Doxycycline (1 μM), Azithromycin (1 μM) plus Vitamin C (250 μM) very potently inhibited CSC propagation by >90%, using the MCF7 ER(+) breast cancer cell line as a model system. The strong inhibitory effects of this DAV triple combination therapy on mitochondrial oxygen consumption and ATP production were directly validated using metabolic flux analysis. Therefore, the induction of mitochondrial biogenesis due to mild oxidative stress, coupled with inhibition of mitochondrial protein translation, may be a new promising therapeutic anti-cancer strategy. Consistent with these assertions, Vitamin C is known to be highly concentrated within mitochondria, by a specific transporter, namely SVCT2, in a sodium-coupled manner. Also, the concentrations of antibiotics used here represent sub-antimicrobial levels of Doxycycline and Azithromycin, thereby avoiding the potential problems associated with antibiotic resistance. Finally, we also discuss possible implications for improving health-span and life-span, as Azithromycin is an anti-aging drug that behaves as a senolytic, which selectively kills and removes senescent fibroblasts.

## Introduction

Previously, we have demonstrated that cancer stem cells (CSCs) critically rely on mitochondrial biogenesis and oxidative metabolism for their propagation [1,2]. Doxycycline, a tetracycline-based antibiotic, is a known inhibitor of the small mitochondrial ribosome (28S) and, as a consequence, is an inhibitor of mitochondrial protein translation [1–4]. Indeed, *in vitro* and *in vivo* evidence supports the potential inhibitory effects of Doxycycline on cancer growth through inhibition of CSC propagation [1–5]. More specifically, we demonstrated that Doxycycline inhibits CSC propagation, as assessed using the 3D mammosphere assay, with an IC-50 between 2-to-10 μM, specifically in MCF7 cells, an ER(+) human breast cancer cell line [1,2]. Importantly, quantitatively similar results were obtained with several other human breast cancer cell lines, such as T47D [ER(+)] and MDA-MB-231 (triple-negative).

Recently, Antibiotic for Breast Cancer (ABC) trial was conducted at The University of Pisa Hospital [5]. The ABC trial aimed to assess the anti-proliferative and anti-CSC mechanistic actions of Doxycycline in early breast cancer patients [5]. The primary endpoint of the ABC trial was to determine whether short-term (2 weeks) pre-operative treatment with oral Doxycycline of stage I-to-III early breast cancer patients resulted in inhibition of tumor proliferation markers, as determined by a reduction in tumor Ki67 from baseline (pre-treatment) to post-treatment, at the time of surgical excision [5]. Secondary endpoints were used to determine if pre-operative treatment with Doxycycline in the same breast cancer patients resulted in inhibition of CSC propagation and a reduction of mitochondrial markers.

A pilot study of the ABC trial demonstrated that Doxycycline treatment successfully decreased the expression of CSC markers in breast cancer tumor samples. Post-doxycycline tumor samples demonstrated a statistically significant 40% decrease in the stemness marker CD44, when compared to pre-Doxycycline tumor samples [5].

CD44 levels were reduced between 17.65% and 66.67%, in 8 out of 9 patients treated with Doxycycline [5]. In contrast, only one patient showed a rise in CD44, by 15%. This represents a nearly 90% positive response rate. Similar results were also obtained with ALDH1 [5], another marker of stemness, especially in HER2(+) patients. In contrast, markers of mitochondria, proliferation, apoptosis and neo-angiogenesis, were all similar between the two groups. These results suggest that Doxycycline can selectively eradicate CSCs in breast cancer patients *in vivo* [5].

Given these promising results in the ABC pilot study, here we aimed to further potentiate the efficacy of Doxycycline, for patient benefit. Our preliminary *in vitro* results indicate that the inhibitory effects of Doxycycline on CSC propagation can be further potentiated, by employing a combination therapy strategy, with two additional pharmacological agents, namely i) Azithromycin and ii) Vitamin C. Azithromycin inhibits the large mitochondrial ribosome, as an off-target side-effect. Vitamin C acts as a mild pro-oxidant, which can produce free radicals and, as a consequence, induces mitochondrial biogenesis.

This combination therapy was designed to stimulate mitochondrial biogenesis, while simultaneously inhibiting mitochondrial protein translation, resulting in functional ATP depletion. This occurs because inhibition of mitochondrial protein translation effectively blocks the production of proteins encoded by mitochondrial DNA (mt-DNA), which are absolutely required for OXPHOS, thereby creating a “rho-zero-like” phenotype. Since Azithromycin is an established inducer of autophagy, this strategy should also stimulate mitophagy, to actively eliminate defective mitochondria. This functional property of Azithromycin may also have implications for aging (see Discussion).

## RESULTS

### Combining two complementary inhibitors of mitochondrial protein translation at a low-dose: Doxycycline and Azithromycin

Here, we experimentally evaluated if the inhibitory effect of Doxycycline on mammosphere formation could be potentiated, by a combination with Azithromycin. To this end, Doxycycline and Azithromycin were tested alone or in combination at low concentrations.

**Figure 1:**
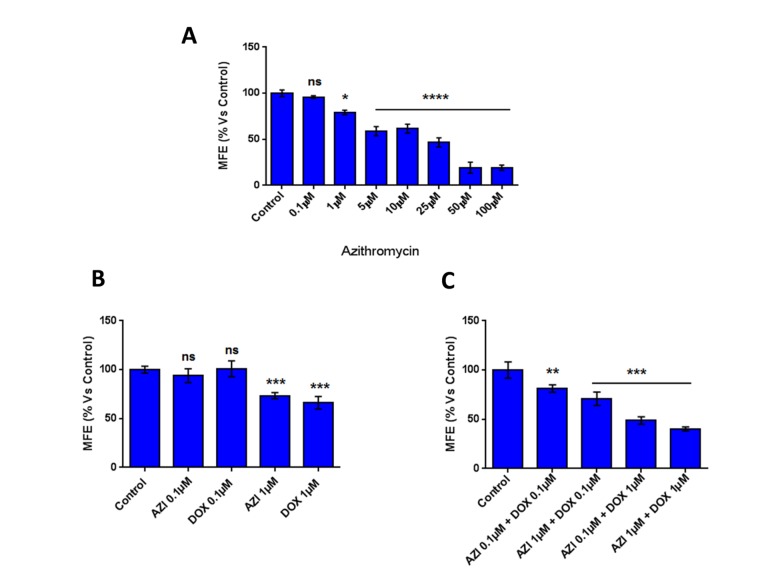
**The combination of low-dose Doxycycline and Azithromycin inhibits mammosphere formation.** Note that this combination in MCF7 breast cancer cells, inhibited 3D mammosphere formation with greater efficacy than the two drugs alone. *p < 0.05; ***p < 0.001; ****p < 0.0001. DOX, Doxycycline; AZI, Azithromycin.

Figure 1 shows that at low concentrations (0.1 μM and 1 μM) Doxycycline and Azithromycin alone had little or no effect on the inhibition of mammosphere formation. However, the combination of 1 μM Doxycycline and 1 μM Azithromycin exerted a very significant inhibitory effect on mammosphere formation.

Note that in combination, Doxycycline and Azithromycin display a marked increased efficacy in the inhibition of mammosphere formation, relative to when the drugs are used alone (the IC-50 for the combination is lower than for Azithromycin alone and lower than for Doxycycline alone, when both agents are used individually). These results suggest that a combination of Doxycycline and Azithromycin might have more therapeutic efficacy, than the two drugs used alone.

**Figure 2:**
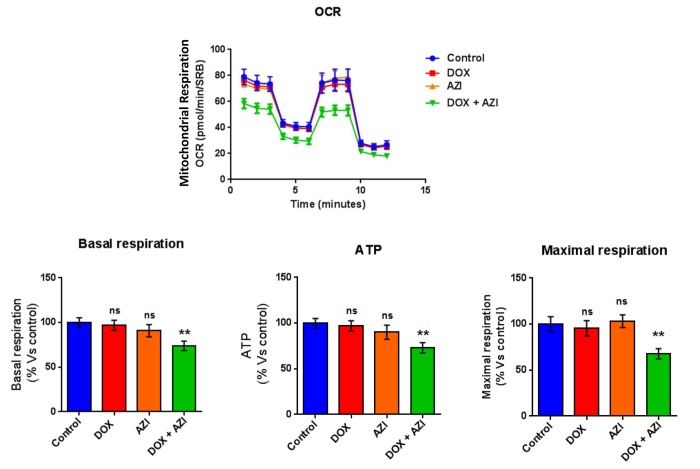
**Combination of low-dose Doxycycline and Azithromycin reduces respiration (basal and maximal) and ATP levels.** The metabolic profile of MCF7 cell monolayers pre-treated with the combination of 1 μM Doxycycline and 1 μM Azithromycin for 3 days was assessed using the Seahorse XFe96 analyzer. **p < 0.01. DOX, Doxycycline; AZI, Azithromycin.

**Figure 3:**
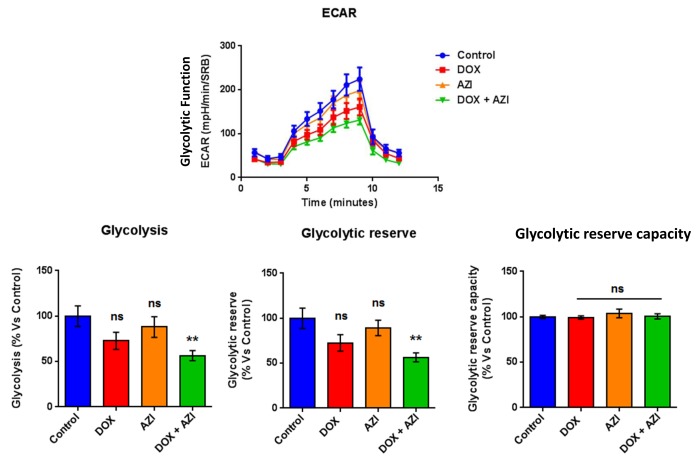
**Combination of low-dose Doxycycline and Azithromycin reduces glycolysis and glycolytic reserve.** The metabolic profile of MCF7 cell monolayers pre-treated with the combination of 1 μM Doxycycline and 1 μM Azithromycin for 3 days was assessed using the Seahorse XFe96 analyzer. **p < 0.01. DOX, Doxycycline; AZI, Azithromycin.

**Figure 4:**
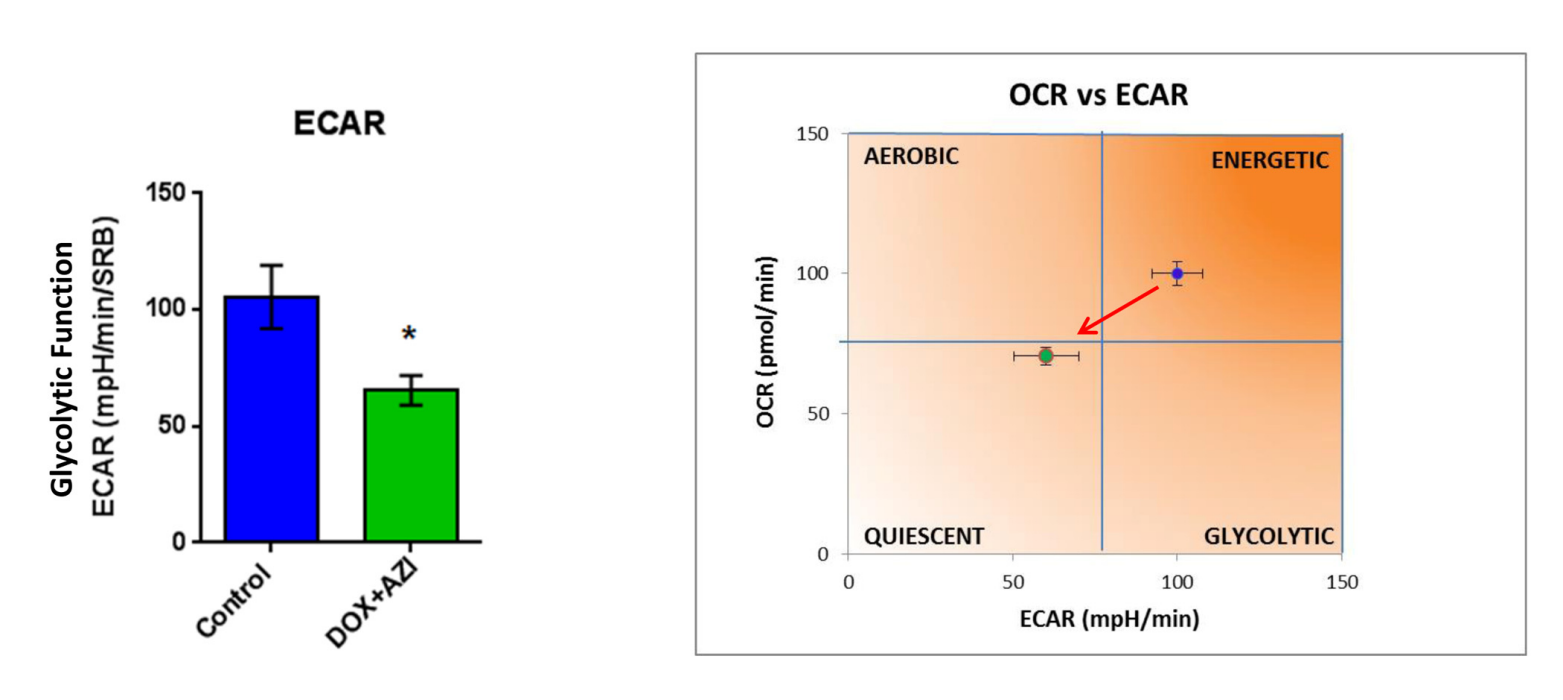
**The Doxycycline-Azithromycin combination shifts MCF7 cancer cells from a highly energetic state to a metabolically quiescent state.** We examined the metabolic profile of MCF7 cell monolayers pre-treated with the combination of Doxycycline (1 μM) and Azithromycin (1 μM) for 3 days, using the Seahorse XFe96 analyzer. *p < 0.05. DOX, Doxycycline; AZI, Azithromycin.

**Figure 5:**
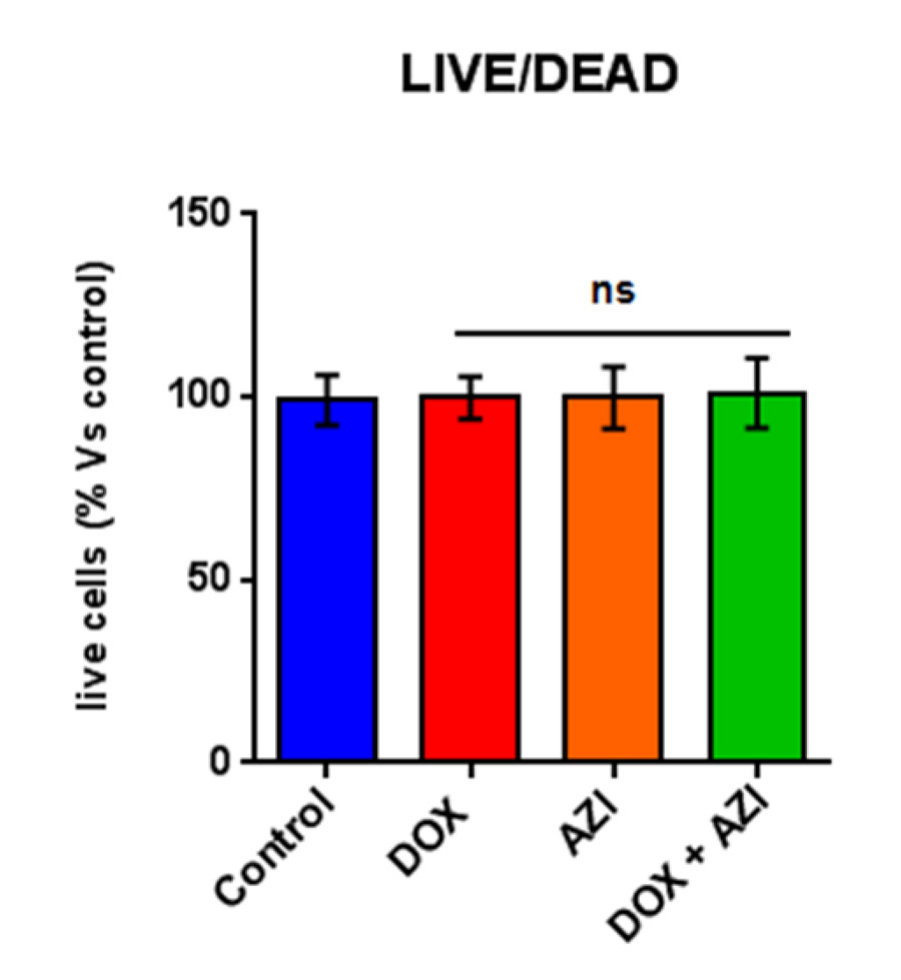
**The Doxycycline (1 μM) plus Azithromycin (1 μM) combination is initially non-toxic under anchorage-independent growth conditions.** MCF7 cells were first treated with the combination for 48 hours, as monolayers, and then they were trypsinized and re-seeded onto low-attachment plates for 12 hours, before they were subjected to a live/dead assay. Note that the combination has no effect on the number of live cells, indicating that it is non-toxic under anchorage-independent growth conditions. Nevertheless, the combination effectively inhibits the propagation of CSCs. DOX, Doxycycline (1 μM); AZI, Azithromycin (1 μM).

To evaluate if the inhibitory effects on mammosphere formation of this double combination is related to mitochondrial function, we next examined the metabolic profile of MCF7 cell monolayers pre-treated with the combination of 1 μM Doxycycline and 1 μM Azithromycin or with the same drugs alone for 3-days. Interestingly, the rates of both oxidative mitochondrial metabolism and glycolysis were significantly reduced by the DOX-AZI combination pre-treatment, as assessed using the Seahorse XFe96 analyzer. This resulted in significant reductions in respiration (basal and maximal), as well as reduced ATP levels (Figure 2). Similarly, both glycolysis and glycolytic reserve were decreased by the DOX-AZI combination (Figure 3). Finally, as seen in Figure 4, MCF7 cancer cells were shifted from a highly energetic profile to a metabolically quiescent state. However, the combination of 1 μM Doxycycline with 1 μM Azithromycin is initially non-toxic under anchorage-independent growth conditions (Figure 5).

Taken together, these results indicate that the combination of low-dose Doxycycline with Azithromycin might be a more efficacious therapeutic option than Doxycycline alone for the eradication of CSCs, by inhibition of mitochondrial function and glycolysis.

### Combining two inhibitors of mitochondrial protein translation, with a pro-oxidant: Doxycycline and Azithromycin Plus Vitamin C (DAV)

Our results indicate that a combination of Doxycycline and Azithromycin is more efficacious than the individual antibiotics in inhibiting CSCs propagation. Thus, we sought to test the hypothesis that a triple combination of Doxycycline, Azithromycin and Vitamin C could more potently inhibit CSC propagation. We have previously shown that Vitamin C alone inhibits CSC propagation with an IC-50 of ~1 mM [6].

**Figure 6:**
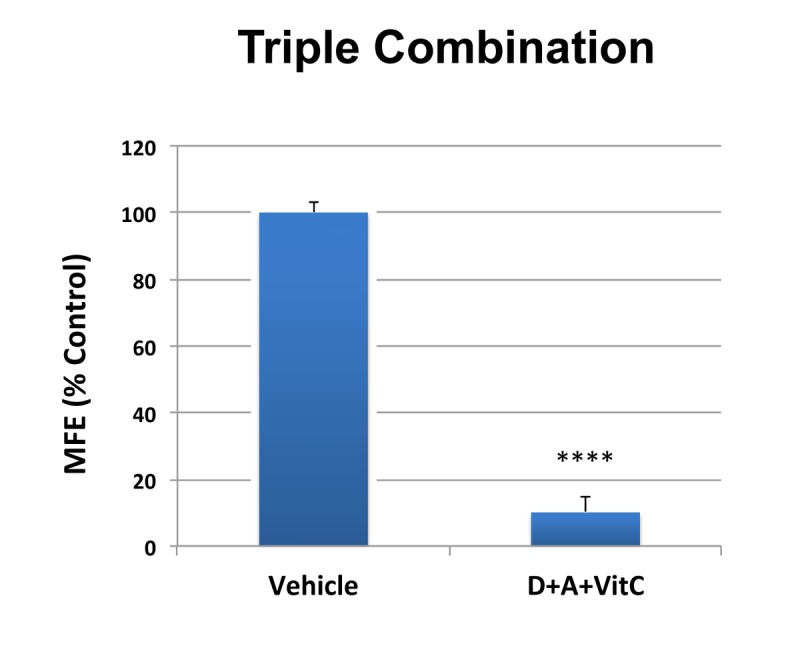
**MCF7 3D mammosphere formation is extremely sensitive to inhibition by combined treatment with Doxycycline [1 μM], Azithromycin [1 μM] and Vitamin C [250 μM] (D+A+VitC).** Bar graphs are shown as the mean ± SD, t-test, two-tailed. Note that approximately 90% inhibition was observed with the triple combination. ****p < 0.0001.

Importantly, simultaneous treatment with 1 μM Doxycycline, 1 μM Azithromycin and 250 μM Vitamin C very potently inhibited CSC propagation by ~90% (Figure 6). Thus, near complete ablation of 3D tumor-sphere forming ability was achieved at very low concentrations of these two antibiotics, suggesting that this DAV triple combination targets CSCs at a vulnerable weak point.

**Figure 7:**
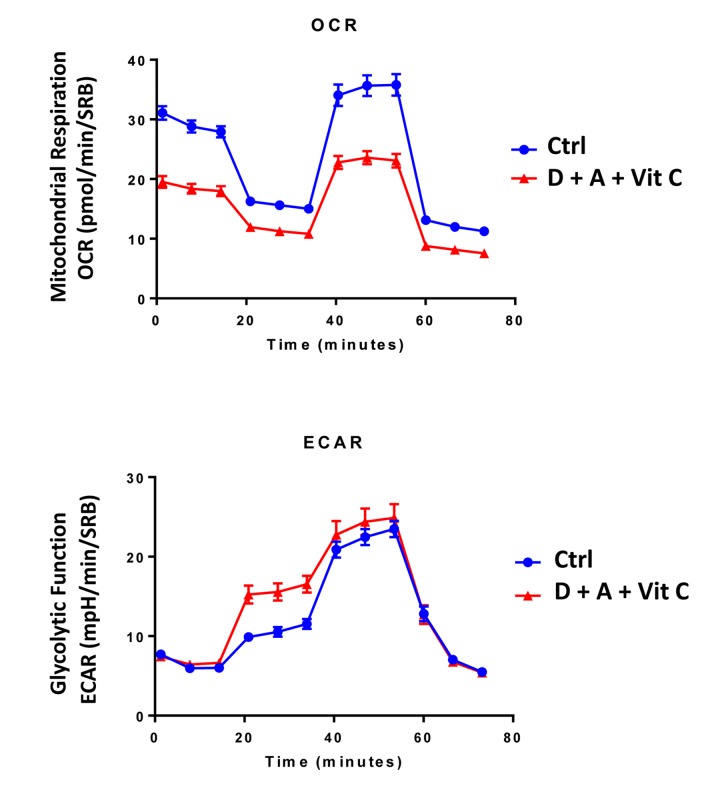
**Combining low-dose Azithromycin and Doxycycline with Vitamin C (DAV), dramatically inhibits metabolism: Seahorse profiles.** The metabolic profile of MCF7 cell monolayers pre-treated with the triple combination (1 μM Doxycycline, 1 μM Azithromycin and 250 μM Vitamin C) for 3 days was assessed using the Seahorse XFe96 analyzer. Note that the DAV triple combination inhibits oxidative mitochondrial metabolism (measured by OCR) and induces glycolytic function (measured by ECAR).

**Figure 8:**
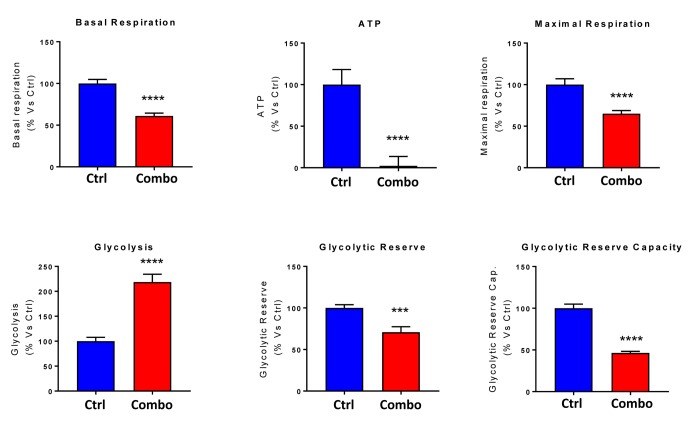
**Combining low-dose Azithromycin and Doxycycline with Vitamin C (DAV), dramatically inhibits metabolism: Bar graphs.** Note that the rate of oxidative mitochondrial metabolism was reduced by >50% and ATP levels were drastically reduced by >95%, as assessed using the Seahorse XFe96 analyzer. This resulted in significant reductions in both basal and maximal respiration. Note also that glycolysis was increased, while glycolytic reserve was decreased by the triple combination. ***p < 0.001; ****p < 0.0001.

Next, we evaluated the potential inhibitory effects of the DAV triple combination on mitochondrial function, by determining the metabolic profile of MCF7 cell monolayers pre-treated with a combination of 1 μM Doxycycline, 1 μM Azithromycin and 250 μM Vitamin C for 3-days. Remarkably, the rate of oxidative mitochondrial metabolism was reduced by >50% and ATP levels were drastically reduced (by >90%), as assessed using the Seahorse XFe96 analyzer. Overall, this resulted in significant reductions in both basal and maximal respiration (Figures 7 and 8). In contrast, glycolysis was increased, while glycolytic reserve was decreased by the DAV triple combination (Figures 7 and 8).

**Figure 9:**
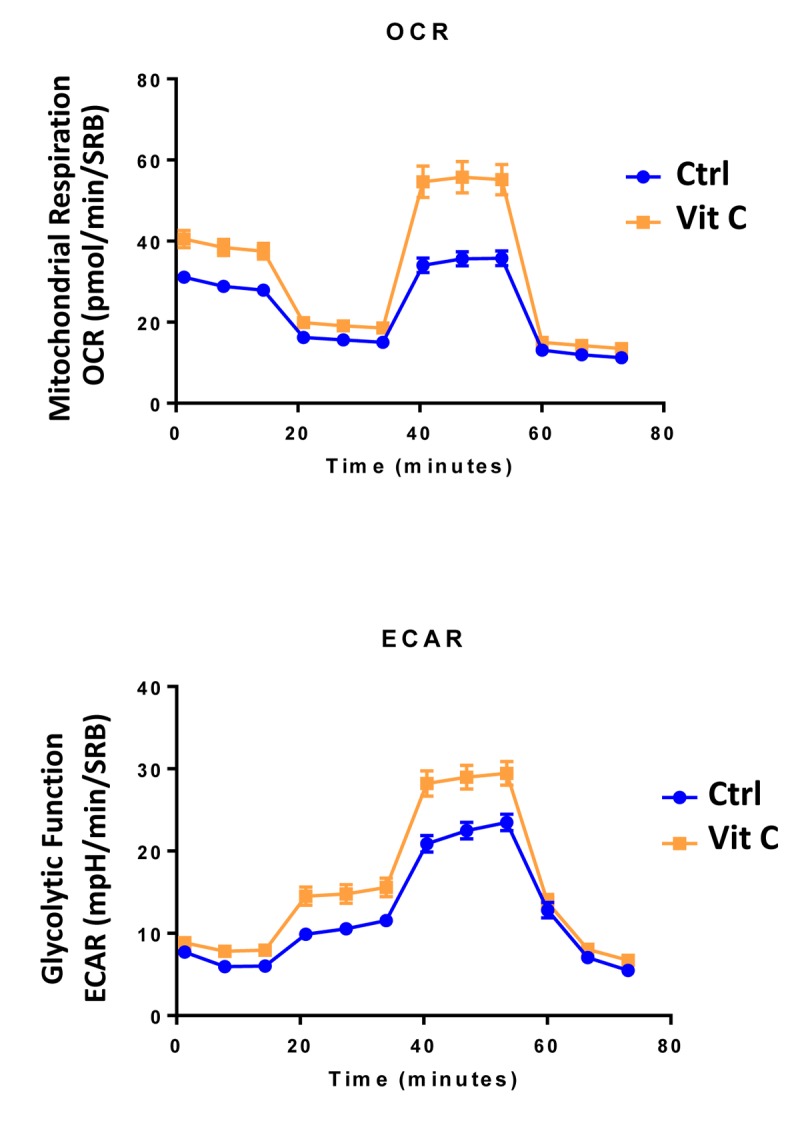
**Low-dose Vitamin C alone increases both mitochondrial metabolism and glycolysis: Seahorse profiles.** The metabolic profile of MCF7 cell monolayers pre-treated with 250 μM Vitamin C alone for 3 days was assessed using the Seahorse XFe96 analyzer. Note that treatment with 250 μM Vitamin C significantly increased both mitochondrial metabolism and glycolysis in MCF7 cancer cells. These observations are consistent with the idea that Vitamin C acts as a mild pro-oxidant and stimulates mitochondrial biogenesis, driving increased mitochondrial metabolism.

**Figure 10:**
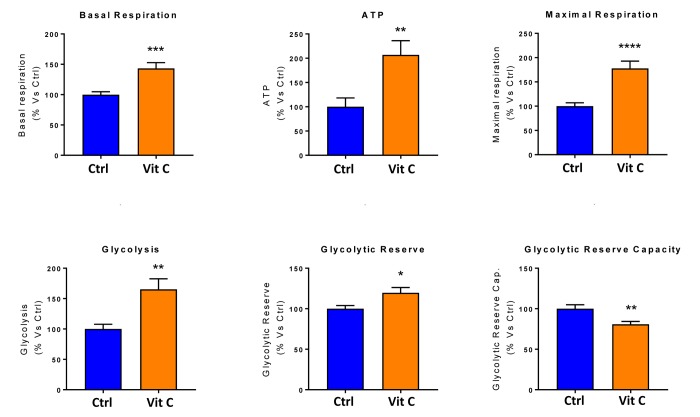
**Low-dose Vitamin C alone increases both mitochondrial metabolism and glycolysis: Bar graphs.** The metabolic profile of MCF7 cell monolayers pre-treated with 250 μM Vitamin C alone for 3 days was assessed using the Seahorse XFe96 analyzer. Note that treatment with 250 μM Vitamin C significantly increased basal respiration, ATP production and maximal respiration. Also, note that treatment with 250 μM Vitamin C significantly increased glycolysis and glycolytic reserves, while decreasing glycolytic reserve capacity. *p < 0.05; **p < 0.01; ***p < 0.001; ****p < 0.0001.

**Figure 11:**
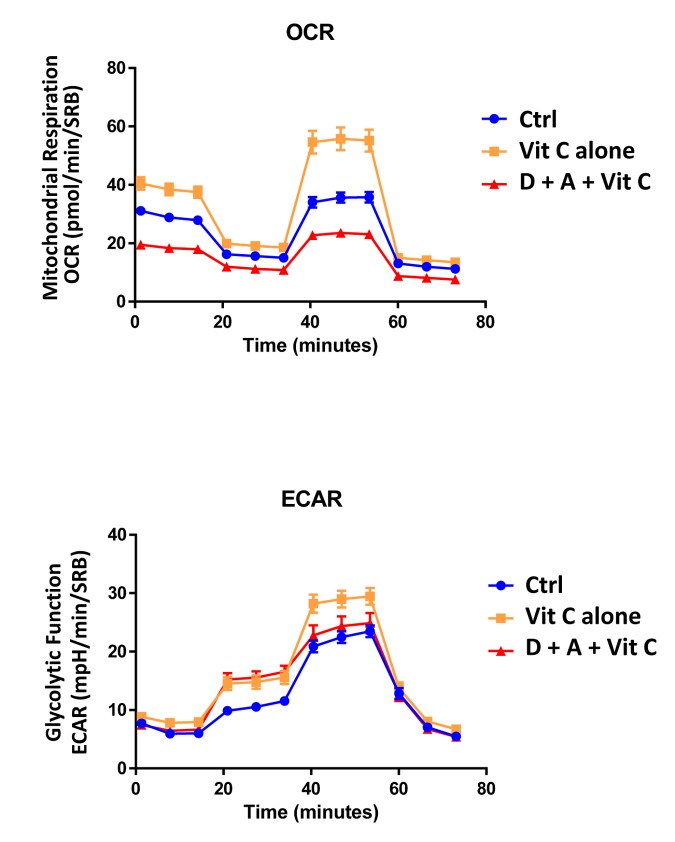
**Direct side-by-side metabolic comparison of low-dose Vitamin C with the DAV triple combination: Seahorse profiles.** Note that low-dose Vitamin C increases oxidative mitochondrial metabolism, while the DAV triple combination results in severe ATP depletion. Also, note that low-dose Vitamin C and the DAV triple combination both increase glycolysis.

**Figure 12:**
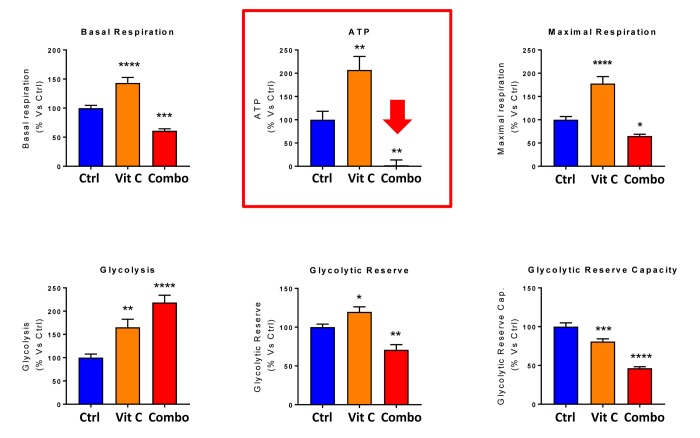
**Direct side-by-side metabolic comparison of low-dose Vitamin C with the DAV triple combination: Bar graphs.** Note that low-dose Vitamin C increases basal respiration, ATP production and maximal respiration, while the DAV triple combination decreases all three of these parameters. Also, note that low-dose Vitamin C and the DAV triple combination both increase glycolysis, while decreasing glycolytic reserve capacity. *p < 0.05; **p < 0.01; ***p < 0.001; ****p < 0.0001.

Finally, as seen in Figure 9 and 10, treatment with 250 μM Vitamin C alone, significantly increased both mitochondrial metabolism and glycolysis in MCF7 cancer cells. These observations are consistent with the idea that Vitamin C alone acts as a mild pro-oxidant and stimulates mitochondrial biogenesis, driving increased mitochondrial metabolism and elevated ATP production. This interpretation is consistent with our experimental data directly showing that the inclusion of two inhibitors of mitochondrial protein translation with Vitamin C, blocks and completely reverses this Vitamin C induced increase in mitochondrial oxidative metabolism (Figures 11 and 12).

Taken together, our evidence supports a novel combined metabolic strategy to better eradicate CSCs. More specifically, we demonstrate that the inhibitory effects of Doxycycline on the CSC population can be potentiated by combination with another FDA-approved antibiotic, Azithromycin, and a dietary supplement, namely Vitamin C (a mild pro-oxidant).

This new DAV therapeutic strategy should provide for the more efficient eradication of CSCs. We aim to test this hypothesis in future clinical trials.

### Evaluating the temporal Effects of pre-treatments on the efficacy of the DAV triple combination, using CSC propagation as a read-out

We devised a system to test whether it was required for all three components of the DAV triple combination to be administered at the same time, by using a pre-treatment strategy, prior to initiating the 3D mammosphere stem cell assay.

Briefly, MCF7 cells, grown as monolayer cultures, were first pre-treated with either Vitamin C alone (250 μM), or Doxycycline Plus Azithromycin (D + A; 1 μM each), for a period of 7 days. Then, MCF7 cells were harvested with trypsin and re-plated under anchorage-independent growth conditions, in the presence of various combinations of Vitamin C, Doxycycline and Azithromycin.

**Figure 13:**
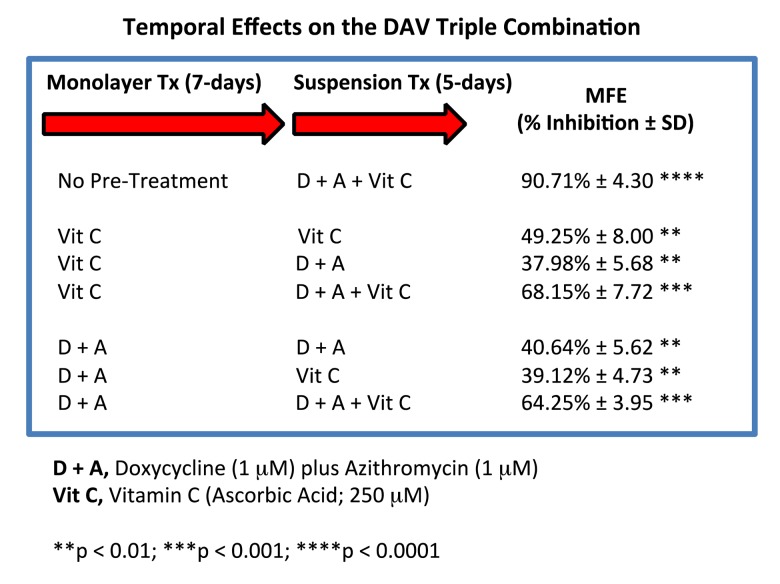
**Effect of various pre-treatments on the efficacy of the DAV triple combination.** Briefly, MCF7 cells, grown as monolayer cultures, were first pre-treated with either Vitamin C alone (250 μM), or Doxycycline Plus Azithromycin (D + A; 1 μM each), for a period of 7 days. Then, MCF7 cells were harvested with trypsin and re-plated under anchorage-independent growth conditions, in the presence of various combinations of Vitamin C, Doxycycline and Azithromycin. Note that 7 days of pre-treatment with either Vitamin C alone or Doxycycline Plus Azithromycin (D + A), rendered the DAV triple combination significantly less effective. Therefore, to achieve maximal impact, we conclude that all three components of the DAV combination of Doxycycline (1 μM), Azithromycin (1 μM) and Vitamin C (250 μM), should be administered together at the same time. MFE, mammosphere formation.

Figure 13 shows that 7 days of pre-treatment with either Vitamin C alone or Doxycycline Plus Azithromycin (D + A), rendered the DAV triple combination significantly less effective. Therefore, it appears that to achieve maximal impact, all three components of the DAV triple combination of Doxycycline (1 μM), Azithromycin (1 μM) and Vitamin C (250 μM), should be administered at the same time.

Mechanistically, it appears that these pre-treatments “pre-conditioned” MCF7 cells to the effects of the DAV triple combination. This may be due to their ability to induce oxidative stress, driving an anti-oxidant response.

## DISCUSSION

**Figure 14:**
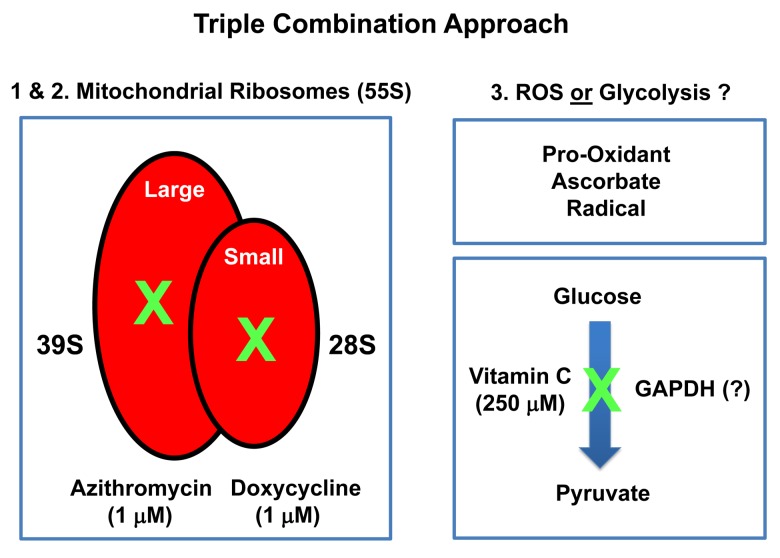
**Summary diagram highlighting the mechanism(s) of action related to the triple combination of Azithromycin, Doxycycline and Vitamin C.** This approach effectively results in the synergistic eradication of CSCs, using vanishingly small quantities of antibiotics. It is important to note Doxycycline and Azithromycin are not direct OXPHOS inhibitors, but instead are inhibitors of mitochondrial protein translation. The 2 metabolic targets are the large mito-ribosome and the small mito-ribosome. Azithromycin inhibits the large mitochondrial ribosome as an off-target side-effect. In addition, Doxycycline inhibits the small mitochondrial ribosome as an off-target side-effect. Vitamin C acts as a mild pro-oxidant and can stimulate the production of free radicals, driving mitochondrial biogenesis, secondary to mitochondrial oxidative stress and the anti-oxidant response. Vitamin C is also thought to act as an inhibitor of the glycolytic enzyme GAPDH (Glyceraldehyde 3-phosphate dehydrogenase). However, here, we did not observe any inhibition of glycolysis, when Vitamin C was tested alone.

In this report, we provide evidence supporting a novel “synthetic-metabolic” approach to target CSCs, via a triple combination therapeutic strategy, which includes two clinically-approved drugs and one essential vitamin. This therapeutic strategy drives the near complete elimination of CSC propagation, but only uses very minute amounts of these compounds. More specifically, this approach involves the simultaneous inhibition of two key targets, namely the large and small mitochondrial ribosomes. Because mitochondria originally evolved from bacteria over 1.45 billion years, they still share certain conserved features related to protein translation. As a consequence, Azithromycin specifically blocks the function of the large mito-ribosome (39S), as an off-target effect. Similarly, Doxycycline inhibits the small mito-ribosome (28S), also as an off-target effect. In contrast, Vitamin C functions as a mild pro-oxidant, producing free radicals. Here, we show that a triple combination of Doxycycline (1 μM), Azithromycin (1 μM) and Vitamin C (250 μM) effectively blocked CSC propagation by ~90% (summarized in Figure 14). In this context, we used the ER(+) breast cancer cell line, namely MCF7, as an established model system for monitoring and quantitating the 3D propagation of CSCs. Finally, we directly validated and confirmed that this DAV triple combination therapy potently inhibited mitochondrial oxygen consumption (OCR) and increased glycolytic flux (ECAR), as predicted. Based on our current metabolic findings, we propose that this triple combination is a feasible and novel anti-cancer strategy for targeting CSCs.

### Cancer: DAV combination therapy for targeting mitochondria in CSCs

**Figure 15:**
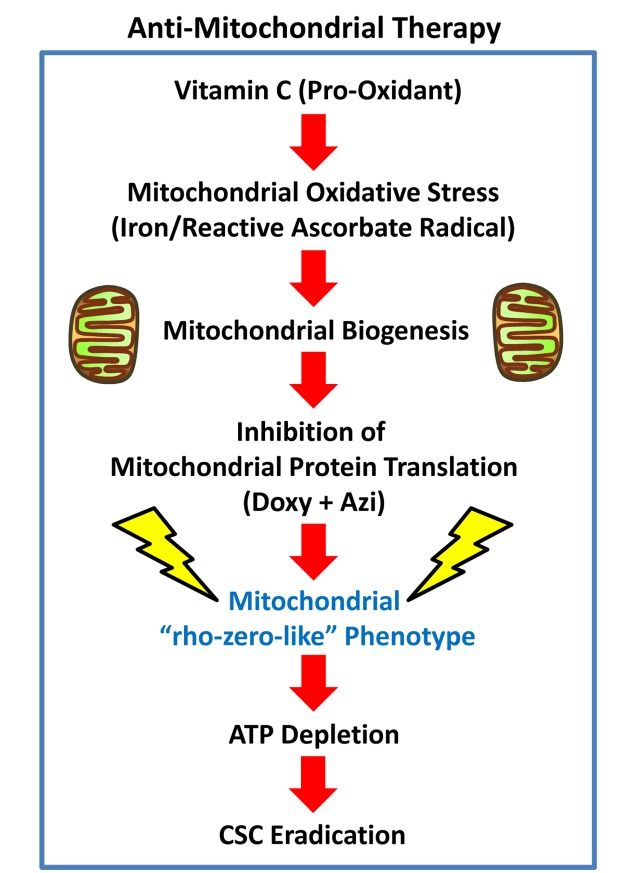
**Anti-mitochondrial therapy.** Vitamin C can act as a pro-oxidant, via the production of free radicals. The ascorbate radical is normally very stable, but becomes highly reactive in the presence of metal ions, including iron (Fe). As mitochondria are rich in iron, they could become a key target of the pro-oxidant effects of Vitamin C, sequentially driving first mitochondrial oxidative stress and then mitochondrial biogenesis. However, the use of inhibitors of mitochondrial protein translation, together with Vitamin C, would ultimately prevent CSC mitochondria from fully recovering, leading instead to CSC eradication. Additional experimentation will be required to further test this hypothesis.

Vitamin C is generally considered to be an anti-oxidant. However, depending on its relative concentration and cellular localization, Vitamin C can also act as a pro-oxidant, via the production of free radicals (Figure 15). The ascorbate radical is normally very stable, but it becomes more reactive especially in the presence of metal ions, including iron (Fe), allowing the ascorbate radical to become a much more powerful pro-oxidant. Therefore, we speculate that as mitochondria are particularly rich in iron, they could become a key target of the pro-oxidant effects of Vitamin C, driving mitochondrial oxidative stress and new mitochondrial biogenesis.

Importantly, Vitamin C is highly concentrated within mitochondria [7–11]. For example, when U937 cells (a human leukaemia cell line) were incubated for only 15 minutes in media containing 3 μM Vitamin C, it was very efficiently transported to the mitochondria, reaching a level of 5 mM – being concentrated ~1,700-fold [8]. Mitochondrial transport of Vitamin C is accomplished by the sodium-coupled Vitamin C transporter 2 (SVCT2) [7–10], also known as SLC23A2, although other novel mitochondrial transporters have been suggested [11].

This mitochondrial targeting of Vitamin C may directly explain the effects we observed here of the triple combination on CSCs, as we have previously shown that CSCs have a significantly increased mitochondrial mass [12,13] and this contributes to their ability to undergo anchorage-independent growth [14,15]. Hence, the use of inhibitors of mitochondrial protein translation, together with Vitamin C, would ultimately prevent CSC mitochondria from fully recovering from the pro-oxidant effects of Vitamin C, as these target cells would be unable to re-synthesize new functional mitochondria (Figure 15). Thus, under these metabolically restricted conditions, cancer cells would be expected to undergo “frustrated” or “incomplete” mitochondrial biogenesis. This assertion is directly supported by the Seahorse flux analysis data shown in Figures 11 and 12, revealing i) reduced mitochondrial metabolism, ii) increased compensatory glycolytic function, and iii) severe ATP depletion.

Based on the mechanism(s) underpinning the strong effectiveness of the triple combination, other pro-oxidants could also be potentially substituted for Vitamin C. As many current chemotherapeutic agents, as well as targeted radiation, all kill cancer cells, via their pro-oxidant actions, then combined inhibition of mitochondrial protein translation could be used as an add-on to conventional therapy and would be predicted to improve their efficacy. However, Vitamin C clearly has fewer side effects and a better safety profile than most standard chemotherapeutic agents. Additional experimentation will be required to further test this intriguing hypothesis.

**Figure 16:**
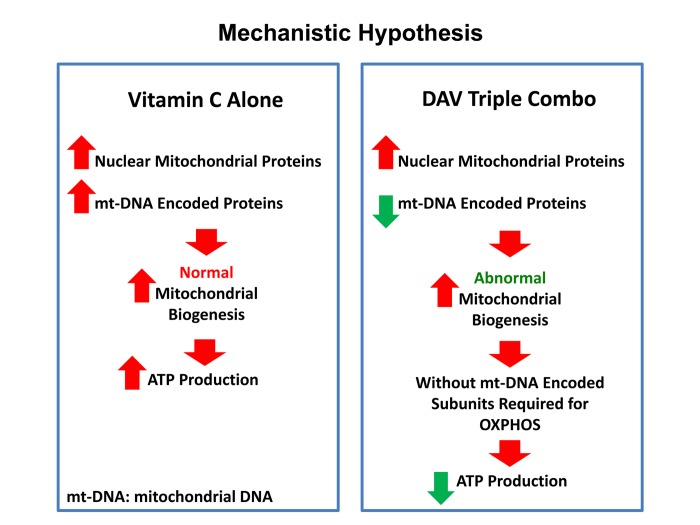
**Vitamin C vs. the DAV triple combination: a mechanistic side-by-side comparison.** *Left panel:* When used as a single agent, Vitamin C can act as a pro-oxidant and induce mitochondrial biogenesis, driving increased mitochondrial protein synthesis and elevated ATP production. *Right panel:* In contrast, the DAV triple combination would preferentially inhibit the synthesis of proteins that are encoded by mitochondrial DNA (mt-DNA), leading to a strict depletion of essential protein components that are absolutely required for maintaining OXPHOS. In the absence of these required OXPHOS components, this would result in abnormal mitochondrial biogenesis and severe ATP depletion. As predicted, we observed dramatic ATP depletion experimentally. Therefore, Vitamin C amplifies the effects of Doxycycline and Azithromycin, by driving mitochondrial biogenesis, thereby diluting out the pre-existing population of mt-DNA encoded proteins. In summary, this strategy was designed to create a “rho-zero-like” phenotype. Also, since Azithromycin is an established inducer of autophagy, this approach should stimulate mitophagy, to actively eliminate defective mitochondria.

Previous studies have shown that Vitamin C by itself increases mitochondrial ATP production by up to 1.5-fold, in the rat heart, under conditions of hypoxia [16]. In addition, Vitamin C is a positive regulator of endogenous L-carnitine biosynthesis, an essential micro-nutrient that is required for mitochondrial beta-oxidation [17,18]. As such, these findings are consistent with our current results showing that Vitamin C alone is indeed sufficient to increase mitochondrial ATP production, by up to 2-fold, in MCF7 cells (Figure 16).

### Aging: Improving health-span and life-span

We believe that the DAV triple combination therapy that we describe here may also have implications for improving health-span and life-span, as aging is one of the most significant risk factors for the development of many human cancer types [19,20]. Moreover, we have previously demonstrated that Azithromycin, by itself, is an FDA-approved drug, with remarkable senolytic activity, that targets and removes senescent fibroblasts, such as myo-fibrobasts, with great efficiency approaching nearly 97% [21]. The accumulation of pro-inflammatory senescent cells is thought to be the primary cause of many aging-associated diseases, such as heart disease, diabetes, dementia and cancer, to name only a few [21]. Since cancer-associated fibroblasts (CAFs) are senescent myo-fibroblasts, with tumor promoting activity, this triple combination approach with Azithromycin may also effectively target the glycolytic tumor stroma of aggressive and metastatic cancers, especially those bearing the metabolic hallmarks of the “Reverse Warburg Effect” [22–28].

## CONCLUSIONS

In conclusion, Phase II clinical trials will be necessary to validate the potential therapeutic efficacy of the DAV triple combination, for eradicating CSCs, in breast cancer patients.

## MATERIALS AND METHODS

### Cell lines and reagents

MCF7 cells, an ER(+) human breast cancer cell line, was originally purchased from the American Type Culture Collection (ATCC), catalogue number HTB-22. Doxycycline, Azithromycin and Ascorbic Acid (Vitamin C) were all obtained commercially from Sigma-Aldrich, Inc.

### Mammosphere formation assay

A single cell suspension was prepared using enzymatic (1x Trypsin-EDTA, Sigma Aldrich, #T3924), and manual disaggregation (25 gauge needle) [12–15]. Cells were plated at a density of 500 cells/cm^2^ in mammosphere medium (DMEM-F12 + B27 + 20 ng/ml EGF + PenStrep) under non-adherent conditions, in culture dishes pre-coated with (2-hydroxyethylmethacrylate) (poly-HEMA, Sigma, #P3932), called “tumor-sphere plates”. Vehicle alone (DMSO) control cells were processed in parallel. Cells were grown for 5 days and maintained in a humidified incubator at 37°C. After 5 days of culture, 3D mammospheres >50 μm were counted using an eye piece (“graticule”), and the percentage of cells plated which formed spheres was calculated and is referred to as percent mammosphere formation efficiency (MFE) and was normalized to one (1 = 100% MFE).

### Metabolic flux analysis

Real-time oxygen consumption rates (OCR) and extracellular acidification rates (ECAR) rates in MCF7 cells were determined using the Seahorse Extracellular Flux (XFe96) analyzer (Seahorse Bioscience, USA) [29–31]. Briefly, 1.5 x 10^4^ cells per well were seeded into XFe96 well cell culture plates, and incubated overnight to allow cell attachment. Then, cells were treated with antibiotics and/or with Vitamin C for 72h. Vehicle-alone control cells were processed in parallel. After 72 hours of incubation, cells were washed in pre-warmed XF assay media (or for OCR measurement, XF assay media supplemented with 10mM glucose, 1mM Pyruvate, 2mM L-glutamine and adjusted at 7.4 pH). Cells were then maintained in 175 µL/well of XF assay media at 37°C, in a non-CO_2_ incubator for 1 hour. During the incubation time, we loaded 25 µL of 80 mM glucose, 9 µM oligomycin, and 1M 2-deoxyglucose (for ECAR measurement) or 10 µM oligomycin, 9 µM FCCP, 10 µM rotenone, 10 µM antimycin A (for OCR measurement), in XF assay media into the injection ports in the XFe96 sensor cartridge. Measurements were normalized by protein content (SRB assay). Data sets were analyzed using XFe96 software and GraphPad Prism software, using one-way ANOVA and Student’s t-test calculations. All experiments were performed in quintuplicate, three times independently.

### Live/dead assay for anoikis-resistance

Following monolayer treatment with either Doxycyline alone, Azithromycin alone or the combination for 48 hours, the CSC population was enriched by seeding onto low-attachment plates [32]. Under these conditions, the non-CSC population undergoes anoikis (a form of apoptosis induced by a lack of cell-substrate attachment) and CSCs are believed to survive. The surviving CSC fraction was then determined by FACS analysis. Briefly, 1 × 10^4^ MCF7 monolayer cells were treated with antibiotics or vehicle alone for 48h in 6-well plates. Then, cells were trypsinized and seeded in low-attachment plates in mammosphere media. After 12h, the MCF7 cells were spun down. Cells were rinsed twice and incubated with LIVE/DEAD dye (Fixable Dead Violet reactive dye; Invitrogen) for 10 minutes. Samples were then analyzed by FACS (Fortessa, BD Bioscence). The live population was then identified by employing the LIVE/DEAD dye staining assay. Data were analyzed using FlowJo software.
